# Genomic and transcriptomic insights into aflatoxin-induced intrahepatic cholangiocarcinoma: an integrated pathway and meta-analysis study

**DOI:** 10.3389/fgene.2026.1785259

**Published:** 2026-03-31

**Authors:** Fengping Li, Yuanyang Ma, Yan Cheng, Shanshan Zou

**Affiliations:** 1 Department of Hepatobiliary Medicine, Third Affiliated Hospital of Naval Medical University, Shanghai, China; 2 Department of Radiology, Mudanjiang Traditional Chinese Medicine Hospital, Mudanjiang, Heilongjiang, China; 3 NHC Key Lab of Reproduction Regulation, Shanghai Institute for Biomedical and Pharmaceutical Technologies, Shanghai, China

**Keywords:** aflatoxin, environmental carcinogenesis, genomic pathways, intrahepatic cholangiocarcinoma, literature data mining, multi-omics integration, transcriptomic meta-analysis

## Abstract

**Introduction:**

Environmental and chemical exposures are major yet incompletely characterized drivers of human carcinogenesis. Aflatoxin, a potent food-borne mycotoxin, has been implicated in tumor initiation, proliferation, and immune suppression in intrahepatic cholangiocarcinoma (ICC), but its genomic mechanisms remain poorly defined.

**Methods:**

We employed extensive literature data mining (LDM) to identify genes associated with both aflatoxin exposure and ICC, enabling construction of a mechanistic genetic pathway linking exposure to disease. These pathways were further evaluated using a meta-analysis of five Gene Expression Omnibus (GEO) expression datasets, followed by functional annotation to characterize their biological roles.

**Results:**

LDM identified 1,754 ICC-associated genes, of which 427 were also linked to aflatoxin, with 154 positioned as potential intermediate regulators connecting aflatoxin exposure to ICC. Meta-analysis revealed significant expression alterations in six genes upon aflatoxin exposure, including upregulation of CRP, CDK2, AXL, and MIR221 (overexpression >50%, p < 0.05) and downregulation of F2 and BUB1B (reduced expression >60%, p < 0.014). Co-expression analysis indicated strong interactions among these regulators (Fisher’s Z > 0.53, p < 0.05), suggesting coordinated molecular responses associated with ICC progression. Functional annotation further highlighted inflammatory responses, cytokine dysregulation, and kinase-related signaling as key processes potentially linking aflatoxin exposure to ICC development.

**Discussion:**

These findings provide a systems-level view of the genomic mechanisms underlying aflatoxin-associated ICC carcinogenesis and identify candidate molecular mediators linking environmental toxin exposure to tumor development. This integrative framework may facilitate exposure-informed biomarker discovery and potential preventive or therapeutic strategies, particularly in regions where aflatoxin exposure remains prevalent.

## 1 Introduction

Intrahepatic cholangiocarcinoma (ICC) is a rare and aggressive form of liver cancer that originates from the bile ducts within the liver, comprising 10%–20% of primary liver cancers globally. The incidence of ICC has been escalating, with risk factors such as chronic liver disease, hepatitis B and C infections, and liver fluke infestations playing significant roles in its pathogenesis (Storandt et al., 2024). Early detection of ICC is challenging due to nonspecific symptoms, and prognosis remains poor primarily due to late diagnosis, limited treatment options, and high recurrence rates post-surgery, influenced by anoikis-resistant cancer stem-like cells (Du et al., 2024; Qin et al., 2024; Yuan et al., 2024). Treatment modalities for ICC include resection, transplantation, chemotherapy, immunotherapy, and targeted therapies, although personalized approaches are being explored for improved outcomes. Factors like tumor size, stage, biomarkers, hepatitis B status, and biliary anatomy impact prognosis in ICC patients, emphasizing the need for tailored interventions and novel therapeutic targets (Fu et al., 2024; He et al., 2024; Ren et al., 2024). Risk factors such as chronic liver diseases, biliary tract disorders, infections, and environmental exposures contribute to the development of ICC, with markers like CDK2 and CRP elevation and immune cell counts influencing both diagnosis and recurrence-free survival (Yamamura et al., 2020; Yugawa et al., 2021).

Aflatoxin, a potent carcinogenic mycotoxin produced by Aspergillus molds in crops like maize and peanuts, has been extensively studied for its detrimental effects on human health. The exposure to aflatoxin is associated with a range of diseases, including liver cancer, hepatitis, and compromised immune function (Dai et al., 2024; Del Fabbro et al., 2024). Chronic ingestion of aflatoxin can result in aflatoxicosis, leading to liver damage, jaundice, and potentially fatal outcomes (Rezaei et al., 2024; Shahdadi et al., 2023). Classified as a Group 1 human carcinogen by the International Agency for Research on Cancer (IARC), aflatoxin poses a significant health risk, necessitating preventive measures and the use of antioxidants to mitigate its toxic effects. Infants are particularly vulnerable to aflatoxin exposure through contaminated breast milk and certain foods, emphasizing the importance of monitoring and regulation to reduce the incidence of aflatoxin-related diseases (Nejad et al., 2023; Palumbo et al., 2023).

A recent investigation has associated aflatoxin with the initiation and proliferation of tumors, as well as immune suppression. This suggests a plausible involvement in the development and advancement of ICC (Dong et al., 2022). Nonetheless, further research is necessary to elucidate the precise mechanisms through which aflatoxin induces carcinogenic effects (Dong et al., 2022). Despite increasing evidence linking aflatoxin exposure to hepatobiliary carcinogenesis, several important gaps remain in understanding its potential role in intrahepatic cholangiocarcinoma (ICC). Most previous studies have focused primarily on experimental toxicology or individual molecular mechanisms, often examining limited sets of genes or pathways. As a result, a systematic framework connecting aflatoxin exposure to ICC-related genomic regulators has not been clearly established. Moreover, existing studies rarely integrate large-scale literature-based knowledge with transcriptomic evidence to validate exposure–gene–disease relationships.

To our knowledge, no previous study has systematically combined literature data mining (LDM), expression-based meta-analysis, and pathway modeling to construct a comprehensive molecular network linking aflatoxin exposure to ICC development. Integrating these complementary approaches allows the identification of candidate molecular mediators supported simultaneously by published knowledge and transcriptomic evidence, thereby providing a more robust systems-level view of exposure-driven carcinogenic mechanisms.

In this study, we conducted an integrated analysis of literature data and expression profiles, aiming to elucidate the genetic pathways connecting aflatoxin to ICC. The findings from our investigation hold promise for shedding light on the potential contribution of aflatoxin to ICC pathogenesis and progression.

## 2 Methods

### 2.1 Study workflow

The workflow proceeded as follows: Initially, a comprehensive literature-based mining endeavor was conducted to gather gene sets associated with ICC and aflatoxin. These gene sets were subsequently compared to ICC-upstream regulators and aflatoxin-downstream targets. Following this, the identified genes underwent validation through a meta-analysis across five publicly available aflatoxin expression datasets sourced from Gene Expression Omnibus (GEO) (https://www.ncbi.nlm.nih.gov/geo/). Subsequently, functional pathway analysis and protein-protein interaction (PPI) through co-expression analysis were performed, leading to conclusions regarding their pathogenic significance in ICC.

### 2.2 Literature-based relation data

Relationships between aflatoxin exposure, intrahepatic cholangiocarcinoma (ICC), and human genes were identified using literature-based data mining. The analysis was performed using the ABT search platform (https://www.gousinfo.com/cnabt/search.html), which systematically analyzes biomedical literature indexed in PubMed and curated ABT databases (https://www.gousinfo.com/en/userguide.html). For each of the 19,924 human genes, literature-supported relationships were evaluated separately for: 1) aflatoxin–gene associations, and 2) ICC–gene associations.

These relationships were identified by scanning PubMed-indexed articles for co-occurrence and contextual associations between genes and the target terms (“aflatoxin” or “intrahepatic cholangiocarcinoma”). Statistical significance of the associations was evaluated using the Adjusted Binomial Algorithm (ABA) [PMID: 40182194], which estimates the likelihood that a gene–term relationship occurs more frequently than expected by chance in the biomedical literature. Gene–term associations with p < 0.01 were considered statistically significant. The resulting relationship tables were then manually curated to remove low-confidence, contradictory, or non-verifiable associations.

Genes that were significantly associated with both aflatoxin exposure and ICC were considered potential intermediate regulators linking aflatoxin to ICC. These overlapping genes were subsequently used to construct the aflatoxin–gene–ICC pathway framework, and were further evaluated using expression-based meta-analysis of aflatoxin-related datasets from the Gene Expression Omnibus (GEO), as described in the following sections.

### 2.3 Data selection for meta-analysis

The relevant expression datasets accessible via GEO were collected through a keyword search for ‘aflatoxin’, leading to the identification of 117 Series data. Subsequently, the following criteria were used to assess the suitability of datasets for this study: i) Organism is *Homo sapiens*; ii) Data type is RNA expression by array; iii) Both dataset and format file are available; and iv) The Study contains both ‘aflatoxin’ cases and normal controls. It is worth noting that while some datasets may include other types of tests, for this study, only cases of ‘aflatoxin’ and their corresponding controls were selected for the meta-analysis, resulting less number of samples. Finally, a total of 5 datasets were considered appropriate for the meta-analysis, as indicated in Table 1.

**TABLE 1:** Five aflatoxin datasets selected for meta-analysis.

GEO ID	Control/Case	Country	Study age	Sample source	Sample organism
GSE25844	10/12	France	15	Hepatocytes	*Homo sapiens*
GSE51175	7/8	Canada	10	Lymphoblastoid cells	*Homo sapiens*
GSE75934	4/3	France	7	Intestinal Caco-2 cells	*Homo sapiens*
GSE87028	4/3	United States of America	7	HepaRG cells	*Homo sapiens*
GSE127791	6/3	United States of America	6	Hepatocytes	*Homo sapiens*

### 2.4 Meta-analysis models

Both fixed-effect and random-effects meta-analysis models were applied to estimate the effect sizes of candidate genes by comparing expression levels between aflatoxin exposure cases and controls across the selected datasets. Gene expression differences were quantified using log2 fold change (LFC) as the effect size.

To determine the appropriate model, heterogeneity among studies was evaluated using the Q statistic and I^2^ index. When the total variance (Q) was less than or equal to the degrees of freedom (df), heterogeneity was considered negligible and a fixed-effect model was applied. Otherwise, a random-effects model was used to account for between-study variability.

Genes were considered significant when both statistical significance and biologically meaningful expression changes were observed. Specifically, the following criteria were applied: 1) p-value <0.05, and 2) absolute expression change exceeding 50%, corresponding to LFC ≥0.59 (≥1.5-fold upregulation), or LFC ≤ −1 (≥50% downregulation). These thresholds were chosen to prioritize genes exhibiting relatively large and biologically meaningful expression changes associated with aflatoxin exposure while minimizing the influence of small expression fluctuations across studies.

### 2.5 Multiple linear regression analysis

To explore whether study-level characteristics influenced the observed gene expression changes associated with aflatoxin exposure, we conducted a multiple linear regression (MLR) analysis. The analysis evaluated the potential contributions of three independent variables—sample size, country of study, and study age—to variations in gene expression.

The dependent variable was the gene expression effect size obtained from the meta-analysis (log_2_ fold change, LFC). The independent variables included: 1) Sample size–defined as the total number of samples (cases and controls combined) in each dataset. This variable was included to account for differences in statistical power across studies. 2) Country of study–datasets were categorized according to the country in which the study was conducted. Countries were encoded using numerical identifiers to allow inclusion as categorical variables in the regression model. This variable was intended to capture potential regional differences that might arise from environmental, population, or experimental factors. 3) Study age–defined as the time interval between the current year and the publication year of the dataset, calculated as: study age = current year − publication year +1. This variable was included to account for possible temporal differences among studies, such as improvements in experimental platforms or analytical methods.

The MLR model was constructed to estimate the relationship between these study-level variables and gene expression changes. For each predictor, the regression analysis produced a regression coefficient (β), p-value, and 95% confidence interval (CI). The regression coefficient represents the direction and magnitude of the association between the predictor variable and gene expression change.

A p-value <0.05 was considered statistically significant. This exploratory analysis was performed to assess whether study-level factors might contribute to variability in gene expression results across datasets.

Because the number of datasets included in the meta-analysis was limited, the MLR analysis should be interpreted as exploratory and intended to identify potential study-level factors that may contribute to heterogeneity.

### 2.6 Functional annotation

For the set of genes identified through expression meta-analysis described above, a functional pathway analysis was conducted with an aim to identify potential biological associations between the selected genes and the ICC. The analysis was performed using the ‘Functional Annotation Tool’ of DAVID (https://david.ncifcrf.gov) against three gene ontologies (GOTERM_BP_DIRECT; GOTERM_CC_DIRECT; and GOTERM_MF_DIRECT) and three pathways (BBID, BIOCARTA, and KEGG_PATHWAY).

### 2.7 Co-expression analysis

A co-expression study was conducted for the significant gene/proteins identified in the LDM/meta-analysis to investigate potential protein-protein interactions (PPIs). Fisher’s Z was utilized as the effect size for the meta-analysis, as depicted in the equation provided (Equation 1).

$$\text{Fisher }Z=0.5\times \log\left(\left(1+\text{Correlation}\right)/\left(1-\text{Correlation}\right)\right)$$ (1)

All co-expression associations exhibiting a P-value <0.05 and Fisher’s Z-value ≥0.5 or ≤ −0.5 were recognized as statistically significant, and subsequently illustrated as a PPI-network.

## 3 Results

### 3.1 Literature-based relation data

Our Literature data mining identified 1754 genes related to ICC supported by about 3,900 references. Among these genes, 427 were also linked to aflatoxin, supported by over 1,000 references. Specifically, 154 out of 427 genes were identified as downstream target of aflatoxin and upstream regulators of ICC, which suggest an aflatoxin → 154 gene → ICC pathway. These 154 genes were further validated using meta-analysis, with results presented in the following sections.

### 3.2 Meta-analysis

**FIGURE 1:**
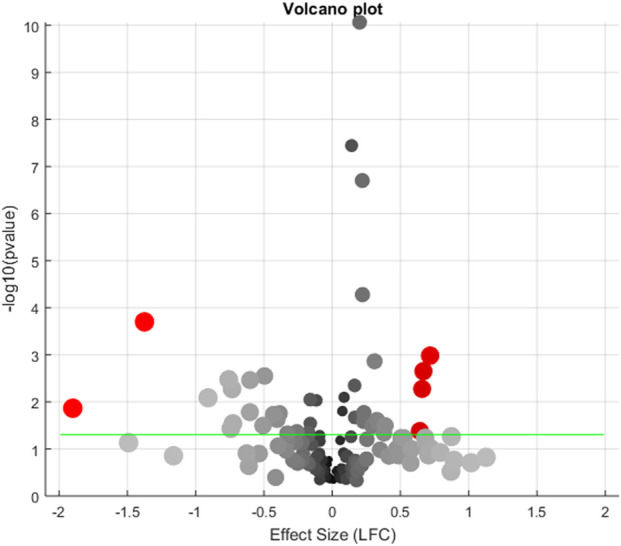
Volcano plot showing the meta-analysis results of 154 genes across 5 aflatoxin expression datasets. Significant genes (LFC >0.59 or LFC < −1; p < 0.05) are highlighted in red, with their names labeled.

For the 154 genes connected aflatoxin and ICC, a meta-analysis has been conducted using 5 aflatoxin expression datasets. Six out of these genes present significant expression change in exposure to aflatoxin, as shown in Figure 1.

The details of the significant genes (LFC>0.59 or < -1; p < 0.05) are presented in Table 2. These genes include *CRP, F2, BUB1B, CDK2, AXL,* and *MIR221*. Among them, *CRP* and *F2* were downregulated by over 60% in exposure to aflatoxin, while *BUB1B, CDK2, AXL,* and *MIR221* were significantly upregulated by over 50%. These findings suggest potential associations between these genes and aflatoxin exposure.

**TABLE 2:** Six significant genes from meta-analysis.

Gene	Meta-analysis results		MLR analysis results (p-value)		
	Effect size (LFC)	p-value	Country	#Sample	Study age
CRP	−1.90	0.014	0.00	0.00	1.00
F2	−1.40	2E-04	0.18	0.011	0.99
BUB1B	0.64	0.042	0.003	1.00	5E-05
CDK2	0.66	0.0050	0.075	0.994	0.0040
AXL	0.67	0.0020	0.027	0.992	0.0050
MIR221	0.72	0.001	—	—	—

It is important to note that genes that partially met our selection criteria—such as those showing high–log10-transformed p-values but minor fold changes in expression—were not considered significant. Including genes with minor expression changes could introduce noise, potentially reducing the robustness of the aflatoxin-driven molecular pathways identified in ICC.

In the MLR analysis, the influence of Country, #Sample, and Study Age on gene expression levels was assessed for *CRP, F2, BUB1B, CDK2, AXL*, and *MIR221*. *CRP* exhibited a significant association with Country (p = 0.00) and #Sample (p = 0.00), but not with Study Age (p = 1.00). *F2* expression was significantly influenced by Study Age (p = 0.011) and moderately by #Sample (p = 0.18), while Country showed no significant impact (p = 0.99). *BUB1B* expression was significantly associated with Country (p = 0.003) and Study Age (p = 5E-05), but not with #Sample (p = 1.00). *CDK2* expression was significantly affected by Study Age (p = 0.075) but not by Country or #Sample (p = 0.994 and p = 0.0040, respectively). *AXL* expression showed significant associations with Study Age (p = 0.027) and #Sample (p = 0.992), but not with Country (p = 0.0050). These findings underscore the diverse influences of these factors on gene expression levels, emphasizing the necessity of considering them in genetic analyses.

**FIGURE 2:**
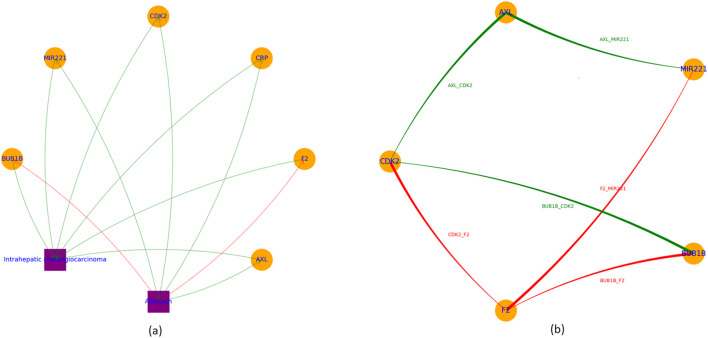
Aflatoxin-driven molecular pathways influencing intrahepatic cholangiocarcinoma. **(a)** Molecular pathways identified through LDM and meta-analysis. **(b)** Protein-protein interaction network generated from co-expression analysis.

The meta-analysis results provide confidence to build the Aflatoxin-driven molecular pathways influencing ICC, as shown in Figure 2a. Interestingly, all these six genes regulated by aflatoxin are promoters of ICC. Therefore, aflatoxin may mainly through the upregulation of four molecules to influence the development and progression of ICC, including *BUB1B, CDK2, AXL*, and *MIR221*.

Co-expression analysis showed that these four genes (*MIR221, CDK2, CRP*, and *AXL*) present strong positive co-expression with each other (Fish’s Z > 0.53, p < 0.05), while significant negative co-expression with F2 (Fish’s Z < 0.70, p < 0.05), as shown in Figure 2b.

### 3.3 Functional annotation analysis

Table 3 summarizes functional annotation results for six aflatoxin-driven ICC regulators, including the term, count, overlap percentage, P-value, and associated genes. It appears that only Gene Ontology (GO) terms reached the significance level (p < 0.05) in the functional annotation analysis conducted using the DAVID Functional Annotation Tool, while other pathways (BBID, BIOCARTA, and KEGG_PATHWAY) did not show significant results at the specified significance level.

**TABLE 3:** Functional annotation results for the six aflatoxin-driven ICC regulators (MIR221, CDK2, CRP, AXL, F2, and BUB1B).

GO term ID	GO term name	Count	Overlap (%)	P value	Overlap genes
GO:0006954	Inflammatory response	3	50	0.005	CRP, MIR221, AXL
GO:0004712	Protein serine/threonine/tyrosine kinase activity	3	50	0.005	AXL, CDK2, BUB1B
GO:0005615	Extracellular space	4	66.67	0.008	CRP, MIR221, AXL, F2
GO:0006953	Acute-phase response	2	33.33	0.011	CRP, F2
GO:1900016	Negative regulation of cytokine production involved in inflammatory response	2	33.33	0.011	MIR221, F2
GO:0046427	Positive regulation of JAK-STAT cascade	2	33.33	0.012	MIR221, F2
GO:0030168	Platelet activation	2	33.33	0.018	AXL, F2
GO:0051897	Positive regulation of protein kinase B signaling	2	33.33	0.047	MIR221, AXL

**FIGURE 3:**
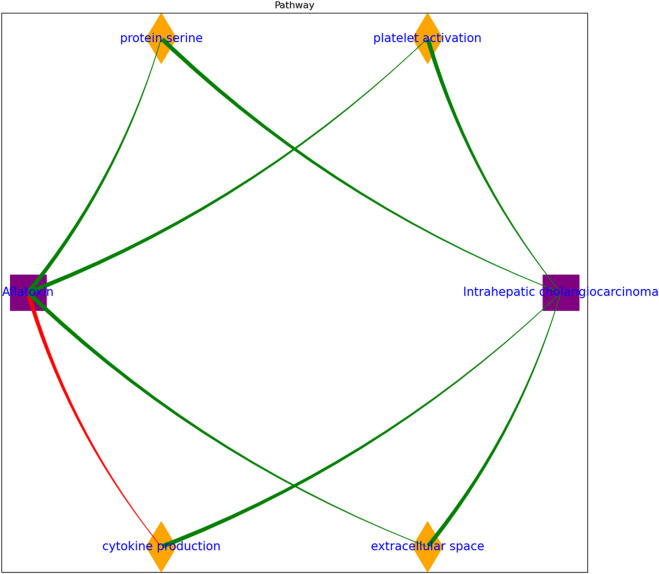
Aflatoxin influences intrahepatic cholangiocarcinoma by regulating multiple cell processes, identified through functional annotation. Genes from the expression meta-analysis were analyzed using the DAVID Functional Annotation Tool to explore their biological associations with ICC.

The functional annotation results also indicate that Aflatoxin exposure influences ICC through a complex interplay of inflammatory responses, cytokine dysregulation, and alterations in cellular processes, as shown in Figure 3. Aflatoxin triggers an acute-phase response and disrupts cytokine production, inducing inflammation and potentially impacting recovery from liver injury. The extracellular space is crucial in both Aflatoxin exposure and ICC, with Aflatoxin causing membrane disruption, oxidative stress, and DNA damage. In ICC, the enhanced extracellular matrix facilitates tumor expansion and invasion. Aflatoxin-induced platelet activation and dysregulated protein kinase B signaling may contribute to ICC progression. Moreover, threonine metabolism is affected in both Aflatoxin exposure and ICC, with potential implications for protein synthesis and cell proliferation. Overall, these findings highlight the multifaceted impact of Aflatoxin on ICC, encompassing various molecular and cellular processes that contribute to tumor development and progression.

## 4 Discussion

Aflatoxin has been shown potential association with the development and progression of ICC, although its mechanisms remain incompletely understood. In this investigation, we developed a provisional molecular pathway through LDM process and validated using expression based meta-analysis. The molecular pathway may partially elucidate the carcinogenic impact of aflatoxin on ICC. Furthermore, functional annotation indicated several cellular process pathways warranting further validation.

Specifically, aflatoxin could promote the activation of four ICC promoters: *MIR221, CDK2, CRP*, and *AXL*. These four genes exhibited over 50% higher expression (p < 0.05) when exposed to aflatoxin, consistent with previous reports (Li et al., 2019; Monmeesil et al., 2019; Uluisik et al., 2023; Valencia-Quintana et al., 2014). For instance, exposure to aflatoxin has been found to upregulate the expression of *MIR221*, which serves as a biomarker for hepatocellular carcinoma in populations exposed to aflatoxin (Valencia-Quintana et al., 2014). Furthermore, *MIR221* promotes ICC progression by regulating cellular pathways, and its overexpression in plasma may aid in the diagnosis of the cancer (Correa-Gallego et al., 2016). This suggests an aflatoxin→*MIR221*→ICC pathway potentially impacting ICC progression and diagnosis.

The influence of aflatoxin on ICC may also involve the upregulation of *CDK2* expression, which plays a significant role in ICC progression by regulating the cell cycle (Li et al., 2019). Inhibition of *CDK2* with Dinaciclib has been shown to reduce proliferation (Saqub et al., 2020), while the expression of *CDK2* is influenced by *Lnc-ATB* and *MiR-200b*, affecting tumor growth (Lin et al., 2019; Yamamura et al., 2020). This evidence suggests an Aflatoxin→*CDK2*→ICC pathway.

Aflatoxin’s influence on ICC may also entail the upregulation of *CRP* levels, thereby impacting inflammation and potentially influencing disease progression (Aborehab and Waly, 2019; Uluisik et al., 2023). In ICC, *CRP* levels are correlated with inflammation, tumor size, *CA19-9* levels, survival rates, and immune response (Gerber et al., 2022; Yugawa et al., 2021). This implies an Aflatoxin→*CRP*→ICC pathway.

Furthermore, aflatoxin may contribute to the progression of ICC through an Aflatoxin→*AXL*→ICC pathway. Aflatoxin upregulates *AXL* expression, and *AXL* overexpression in ICC drives tumor progression via signaling pathways, indicating *AXL* as a potential therapeutic target (Monmeesil et al., 2019; Zhang C. et al., 2024).

Beyond the individual gene-level observations, the identified regulators—CDK2, AXL, MIR221, and CRP—suggest a convergent mechanistic framework linking aflatoxin exposure to ICC development. CDK2 plays a central role in cell-cycle regulation, promoting the transition from the G1 to S phase and facilitating uncontrolled proliferation in tumor cells (PMID: 19238148). AXL, a receptor tyrosine kinase of the TAM family, activates multiple oncogenic signaling pathways, including PI3K–AKT and MAPK, which enhance cell survival, migration, and resistance to apoptosis (PMID: 28072762). MIR221 is known to function as an oncogenic microRNA that regulates cell-cycle inhibitors and promotes tumor growth and invasion. Together, these molecules may represent interconnected components of a broader network involving cell-cycle dysregulation, oncogenic signaling activation, and inflammatory responses. In addition, CRP reflects systemic and local inflammatory activation, which is increasingly recognized as a key driver of tumor microenvironment remodeling in hepatobiliary malignancies (PMID: 34117554).

From a mechanistic perspective, these findings suggest that aflatoxin exposure may contribute to ICC development through multiple interacting processes. Aflatoxin is known to induce DNA damage and mutagenic DNA adduct formation, as well as oxidative stress in hepatocytes (PMID: 19875698; PMID: 22981896). At the same time, aflatoxin exposure can activate inflammatory signaling pathways and immune responses within the liver microenvironment (PMID: 38309422). These processes may collectively promote genomic instability, dysregulated cell proliferation, and tumor-promoting inflammatory conditions. Therefore, the aflatoxin–gene–ICC pathways identified in this study likely reflect a combination of hepatocyte-driven oncogenic signaling and inflammation-mediated tumor microenvironment alterations, providing a systems-level perspective on the molecular mechanisms linking environmental toxin exposure to ICC carcinogenesis.

Functional annotation of the genes mentioned above also triggers the investigation of cell processes in which they are involved, potentially elucidating the mechanisms underlying the carcinogenic impact of aflatoxin on ICC. As illustrated in Figure 3, aflatoxin’s influence on ICC may involve multifaceted mechanisms in regulating the extracellular space. Aflatoxin disrupts cell membranes, resulting in oxidative stress and DNA damage in neighboring cells (Wang et al., 2018). The extracellular environment within ICC significantly influences tumor progression, metastasis, and response to therapy (Watakulsin et al., 2023; Yoshimoto et al., 2023), emphasizing its pivotal role as a potential therapeutic target and diagnostic indicator.

Moreover, aflatoxin exposure has been linked to platelet activation, which exacerbates inflammation and thrombosis. Specifically, Aflatoxin B1 interacts with platelets through protein kinase C activation and phosphatidylinositol-4,5-biphosphate hydrolysis (Muriel, 2009; Van den Heever and Dirr, 1991). In the context of ICC, platelet activation plays a pivotal role in tumor progression by promoting angiogenesis, immune evasion, and cell migration (Yoshimoto et al., 2023). Therefore, Aflatoxin-induced platelet activation likely contributes to the advancement of ICC by facilitating these mechanisms.

The influence of aflatoxin on ICC may also occur through the inhibition of protein kinase B (AKT) signaling. Aflatoxin disrupts AKT signaling, which can impair cell growth and metabolism, potentially contributing to cancer development (Wang et al., 2024; Zhang, F. L. et al., 2024). Additionally, aflatoxin exposure leads to the phosphorylation of protein serine, which may contribute to the promotion of liver cancer, including ICC (Ruggeberg et al., 2020; Ye et al., 2020), as protein serine impacts cell processes, tumor growth, and metastasis in ICC (Zhang et al., 2023).

In addition, Aflatoxin exposure disrupts cytokine production, as evidenced by a previous study (Bastos-Amador et al., 2023). This disruption in cytokine production has significant implications for immune responses and inflammation. ICC, on the other hand, is influenced by cytokine production, affecting tumor progression, immune response, and therapy response (Chang et al., 2024; Salao et al., 2023).

In summary, our findings suggest that aflatoxin exposure may lead to the upregulation of genes such as *MIR221*, *CDK2, CRP,* and *AXL*, all of which have been associated with ICC progression and diagnosis. These genes, along with the pathways and cellular processes they are involved in, provide insights into the mechanisms underlying aflatoxin-induced ICC carcinogenesis. These processes further underscore the complex nature of aflatoxin-mediated ICC progression and provide potential avenues for therapeutic intervention and diagnostic development.

Several limitations of this study should be acknowledged. First, we did not evaluate the association between the expression of the identified genes (MIR221, CDK2, CRP, and AXL) and clinical outcomes in ICC patients using independent cohorts such as TCGA, which would further support their potential prognostic relevance. Second, the meta-analysis was based on five publicly available GEO datasets with relatively small sample sizes, which may limit statistical power. Moreover, these datasets were derived from heterogeneous cell types (e.g., hepatocytes, lymphoblastoid cells, intestinal epithelial cells, and HepaRG cells), potentially introducing biological variability when interpreting the results in the context of ICC. Third, literature-based data mining may be influenced by publication bias toward well-studied genes, which could affect the identification of candidate regulators. Finally, the molecular pathways proposed in this study were inferred from computational analyses integrating literature evidence with aflatoxin exposure datasets rather than transcriptomic data directly obtained from ICC tissues. Therefore, the identified exposure–gene–ICC relationships should be interpreted as candidate mechanistic links requiring further validation in disease-specific datasets and experimental models. Despite these limitations, the integrative framework presented here provides a systematic strategy for identifying potential molecular mediators linking environmental toxin exposure to ICC development.

## 5 Conclusion

In conclusion, through a combination of literature data mining, pathway analysis, and expression-based meta-analysis, we have identified several key genes and cellular processes implicated in the carcinogenic impact of aflatoxin on ICC. Our investigation highlights the complex nature of aflatoxin-mediated ICC progression and offers potential targets for therapeutic intervention and diagnostic development. Further research is needed to validate these findings and explore additional pathways involved in aflatoxin-induced ICC pathogenesis.

## Data Availability

The original contributions presented in the study are included in the article/supplementary material, further inquiries can be directed to the corresponding author.
